# Does Electrode Spacing Truly Control Capacitance and Energy Density in Graphene-Based Supercapacitors? A Molecular Simulation Perspective

**DOI:** 10.1021/acsomega.6c02890

**Published:** 2026-05-26

**Authors:** Davi de Oliveira Guedes, Henrique de Araujo Chagas, Iuliia V. Voroshylova, Guilherme Colherinhas

**Affiliations:** † Instituto de Física, 67824 Universidade Federal de Goiás, Goiânia, Goiás 74690-900, Brazil; ‡ REQUIMTE LAQV, Department of Chemistry and Biochemistry, Faculty of Sciences, 131674 University of Porto, Porto 4169-007, Portugal

## Abstract

Electrode spacing is frequently considered a parameter in supercapacitor optimization, yet its intrinsic impact on capacitance and energy storage under nanoconfinement remains uncertain. In this study, classical molecular dynamics simulations were performed for graphene-based supercapacitors containing the ionic liquid [emim]­[ala] confined between planar electrodes separated by 4–12 nm. Electric potential profiles enabled the calculation of differential and total capacitances as well as stored energy densities. Mass density analyses show well-defined electric double layers (EDLs) at both electrodes. Strong overlap is observed at short separations, whereas a bulk-like central region is preserved at larger ones. Nevertheless, the total capacitance remains nearly constant (∼2.40–2.58 μF/cm^2^), and the differential capacitance at zero charge varies only slightly across all separations, indicating a surface-dominated storage mechanism. The integrated areal energy density up to 2.5 V is also essentially independent of spacing (∼7.4–7.7 μJ/cm^2^). In contrast, gravimetric and volumetric energy densities decrease with increasing separation due to mass and volume normalization effects. These results demonstrate that electrode spacing modulates EDL structure but only weakly affects intrinsic capacitive performance.

## Graphical abstract

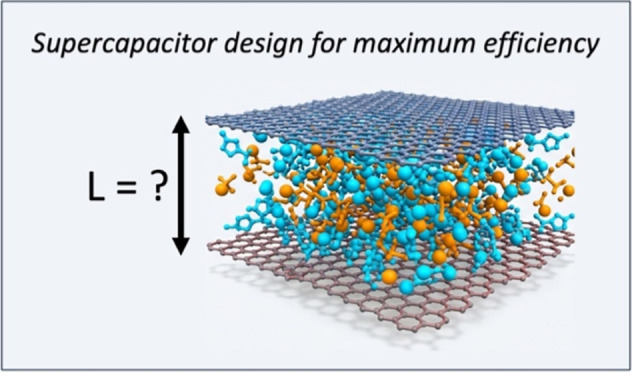

## 1 – Introduction

Understanding the relationship between electrode spacing and the formation of the electric double layer (EDL) in supercapacitors is fundamental for the advancement of electrochemical energy storage. Since the early theoretical developments, such as the extension of Poisson–Boltzmann theory presented by Attard, Mitchell, and Ninham in 1988,^1^ it became evident that the classical description of the EDL was insufficient to adequately represent ionic correlation effects and image charge influences in confined systems. These authors demonstrated that, at large separations, corrections to Poisson–Boltzmann theory manifest as an effective surface charge, while at small distances, the interaction between charged surfaces requires a more refined treatment. This initial point already indicated that electrode spacing could not be treated merely as a geometric parameter but rather as a determinant factor in modulating capacitive properties and interfacial forces in confined electrolytes.

More recent studies, such as the doctoral thesis of Sitlapersad (2023),^2^ broadened this framework by investigating, through molecular simulations, different methodologies to control potentials and charges in supercapacitor electrodes. The author demonstrated that replacing Gaussian charge distributions with point-charge representations within the constant potential method generates significant gains in scalability and accuracy. By developing the generalized constant potential method (GCPM) and the constant sum-charge method (CSCM), the study revealed that the behavior of capacitance and the dynamic response of the supercapacitor critically depend on how the system is modeled as a function of electrode separation. Thus, the thesis reinforces that electrode spacing cannot be dissociated from the methodological approach used to describe EDL dynamics.

The paradigmatic nature of this discussion was clearly established by Kornyshev in 2007,^3^ who argued that ionic liquids should not be analyzed through classical electrochemical theories of dilute solutions. The author introduced a model based on finite-volume exclusion and ionic correlation effects, showing that, in confined ionic liquids, capacitance may exhibit a maximum near the potential of zero charge, in contrast with the minimum predicted by the Gouy–Chapman law. Most relevant for the present discussion, Kornyshev emphasized that electrode spacing drastically modifies capacitance behavior, especially when the distance is comparable to the ionic diameter. Thus, capacitance as a function of spacing is not monotonic but rather subject to structural oscillations that directly reflect the organization of the EDL under confinement.

The comprehensive reviews by Jeanmairet, Rotenberg, and Salanne (2022)^4^ consolidated these perspectives, highlighting how microscopic simulations allow characterization of adsorption, dynamics, and charging mechanisms in nanoporous electrodes. The authors showed that electrode spacing, and more specifically the nanoconfinement regime in pores and slits, determines phenomena such as two-dimensional phase transitions, anomalous charging, and ionic interactions across pores. They also reinforced that optimization of supercapacitor properties requires understanding how the overlap or independence of EDLs, modulated by electrode separation, directly affects capacitance and device power. This body of evidence links the microscopic to the macroscopic scale, showing that classical geometric variables such as cell thickness must be reinterpreted in light of molecular-level structure.

In this context, spectroscopic and impedance analyses also play a crucial role. The work of Mei et al. (2018)^5^ made essential contributions by providing physical interpretations of Nyquist plots in electric double-layer supercapacitors. Through transport simulations and experimental validation, the authors demonstrated that resistances associated with the EDL and ionic transport in the electrolyte can be directly extracted from impedance curves without relying on simplified equivalent circuits. This advanced interpretation reveals that electrode spacing not only controls EDL overlap but also modulates ionic transport and therefore influences the internal resistance and energy dissipation of the device.

Experimental studies on graphitic materials with X-ray-determined interlayer spacing have also shown that structural spacing can affect electrochemical behavior. For example, partially reduced graphite oxide electrodes with interlayer distances in the 0.33–0.46 nm range exhibited spacing-dependent activation and capacitance in organic electrolyte media.^6^ However, such systems are not directly equivalent to the present model, since they involve subnanometric interlayer spacing within a lamellar carbon material, together with electrochemical activation/intercalation effects, rather than the electrode–electrode separation in a planar graphene slit geometry containing [emim]­[ala]. Therefore, these experimental results should be viewed as qualitatively supporting the broader relevance of spacing effects, but not as a direct side-by-side validation of the simulations reported here.

More recent reviews have further expanded this discussion. Kondrat, Feng, Bresme, Urbakh, and Kornyshev (2023)^7^ analyzed in depth the behavior of ionic liquids confined in nanopores, highlighting phenomena such as superionic states, capacitance oscillations, and electrotunable friction. Cats, Sitlapersad, den Otter, Thornton, and van Roij (2022),^8^ in turn, demonstrated the excellent agreement between brownian dynamics simulations and density functional theory (DFT)^9,10^ calculations for planar electrodes, emphasizing the need to correctly account for ensemble conditions in order to reproduce experimental results. Both studies underscore that electrode spacing is a determining parameter in distinguishing regimes of independence versus overlap of the EDL. Complementarily, these works point out that optimizing supercapacitor performance requires the integration of advanced statistical models, molecular simulations, and experimental analyses of dynamic response, all anchored in rigorous control of electrode separation.

In recent molecular dynamics (MD) studies applied to supercapacitors, a convergent computational practice has emerged: fixing the electrode spacing within an intermediate nanometric range, typically around 10–12 nm, while varying other variables (ionic composition, degree of hydration, applied potential, electrode topology) to isolate the impact of confinement without incurring spurious interactions from periodic images. Studies with hydrated ionic electrolytes on graphene electrodes show that capacitive performance and EDL organization are extremely sensitive to this separation scale, employing MD cells with fixed “nanogaps” when comparing anions (Cl^–^/Br^–^) and the electrochemical response of the system, thereby keeping the degree of EDL overlap constant while investigating effects of composition and voltage.^11^ Related works on ionic liquids and mixtures (including natural deep eutectics) report analogous protocols in which the electrode–electrode slit width remains constant on the order of 10 Å, ensuring comparability across simulation series and allowing researchers to distinguish regimes of nearly independent versus partially overlapping EDLs.^12^ In parallel, investigations focused on anion composition in hydrated ILs (bmim-based)^13^ emphasize that maintaining the gap fixed while varying electrolyte chemistry is crucial to correctly attribute changes in differential capacitance and ionic transport to EDL structure, rather than to geometric alterations of confinement. This strategy also appears in performance analyses with graphene/graphyne-based electrodes, where the use of fixed nanometric separations facilitates direct comparison of electrostatic properties, charge dynamics, and energy/power metrics across 2D carbon architectures.^14,15^ Recent reviews and feature articles on graphene-based materials for supercapacitors reinforce the rationale of this methodological choice, highlighting that intermediate nanogaps (∼10–12 nm) balance two requirements: avoiding extreme confinement (which introduces dominant structural oscillations) and at the same time preserving a measurable interaction between EDLs needed to discuss capacitance optimization and resistive losses.^16^ Taken together, these contributions consolidate a simulation design standard in MD, in which the electrode–electrode distance is kept fixed at ∼10–12 nm, providing a robust control axis to elucidate intrinsic EDL trends under chemical and operational variations.

Recent molecular dynamics studies have reinforced the role of graphene as a robust benchmark electrode for elucidating electric double-layer structure in ionic-liquid supercapacitors.^17^ In particular, simulations of amino acid ionic liquids at planar graphene electrodes have shown that, although the positively charged interface is sensitive to the chemical nature of the amino acid anion, the resulting differential capacitance–potential curves and overall capacitances remain comparable to those of conventional ionic liquids. More recent graphene-based studies with biodegradable amino acid ionic liquids further showed capacitances in the range of 2.29–2.71 μF/cm^2^ ^18^ and demonstrated that controlled hydration can preserve or even improve interfacial ordering and energy storage.^19^ These results support the broader view that amino acid ionic liquids constitute viable and efficient electrolytes for sustainable graphene-based supercapacitors.^20^

From a methodological perspective, previous comparisons between constant charge and constant potential simulations show that the suitability of each approach depends strongly on the interfacial regime under investigation. Early work demonstrated that fixed-charge electrode models may alter the local adsorbed structure and, more markedly, transient charging dynamics when compared with constant-potential descriptions.^21^ However, later studies at flat or relatively open ionic-liquid/electrode interfaces reported very similar differential capacitances and adsorbed-ion structures for the two approaches, indicating that constant charge can reproduce the main equilibrium EDL trends in planar systems.^22^ More recent analyses have formalized this picture by showing that the constant charge method is often adequate for equilibrium EDL studies in open electrode systems with ionic liquids, whereas constant potential becomes essential in strongly nanoconfined metallic nanopores, low-density quasi-two-dimensional pores, or voltage-driven charging problems.^23,24^ Within this framework, the present use of the constant charge model should be understood as a computationally efficient first-order description of equilibrium spacing effects in a planar graphene supercapacitor.

Although practical supercapacitors are macroscopic devices with complex porous architectures, the atomistic objective of the present model is to isolate the local interfacial physics of electric double-layer formation under controlled confinement. In this sense, nanoscale slit models are not intended to reproduce the full geometry of a real device, but rather to provide a mechanistic framework for analyzing ion layering, EDL overlap, and intrinsic capacitance trends at the electrode–electrolyte interface. This strategy is consistent with current molecular dynamics practice,^25–27^ in which fixed nanometric separations are commonly employed to probe interfacial electrostatics while minimizing spurious periodic-image effects. Accordingly, the present model spans a confinement regime wide enough to distinguish between strongly overlapping EDLs and systems in which a bulk-like central region can be recovered. For larger device dimensions, EDL overlap would be expected to decrease further, but additional structural factors absent from the present model, such as pore-size distribution, tortuosity, roughness, defects, and long-range ion transport through porous electrode networks, may become relevant for practical device-level performance. Therefore, the present simulations should be interpreted as a nanoscale mechanistic benchmark for intrinsic interfacial behavior rather than as a full geometrical representation of a commercial supercapacitor.

Against this background, it becomes clear that analyzing the behavior of capacitance and other electrochemical properties as a function of electrode spacing is a question that requires detailed investigation at the atomic scale. Although the articles discussed provide a solid foundation, there remains a need for systematic studies combining theory and simulation. In this context, the present article proposes performing classical molecular dynamics simulations of systems containing planar electrodes and confined electrolytes. This approach will allow direct evaluation, over time, of EDL overlap, structural fluctuations, and the impact of electrode spacing on differential capacitance, ionic transport, and equivalent series capacitance. The study to be developed will provide original results, deepening the observations already reported in the literature and enabling the proposal of new optimization protocols for nanoconfined supercapacitors.

## 2 – Methodology

### 2.1 System Setup

In this work, five supercapacitor models were constructed with electrode–electrode separations (*L*) of 4, 6, 8, 10, and 12 nm. Each system consisted of two parallel graphene electrodes confining the ionic liquid electrolyte 1-ethyl-3-methylimidazolium alaninate ([emim]­[ala]), as depicted in Figure 1. Each graphene electrode consisted of 540 carbon atoms and had lateral dimensions of 3.7080 × 3.8529 nm^2^, corresponding to a fixed surface area of 14.287 nm^2^. The positive and negative electrodes were represented in blue and red, respectively, while [emim] cations are shown in cyan and [ala] anions in orange. The molecular systems were generated using Packmol^28^ for initial packing, Gromacs 2023 for molecular dynamics (MD) simulations,^29,30^ and visual molecular dynamics (VMD) for visualization and analysis,^31^ together with in-house tools. Graphene electrodes were modeled using the CHARMM36 force field,^32,33^ while the ionic liquid was parametrized according to the force field proposed in.^34–36^ Table 1 shows the system composition and Figure 1 shows examples of the systems simulated systems.

**Figure 1:**
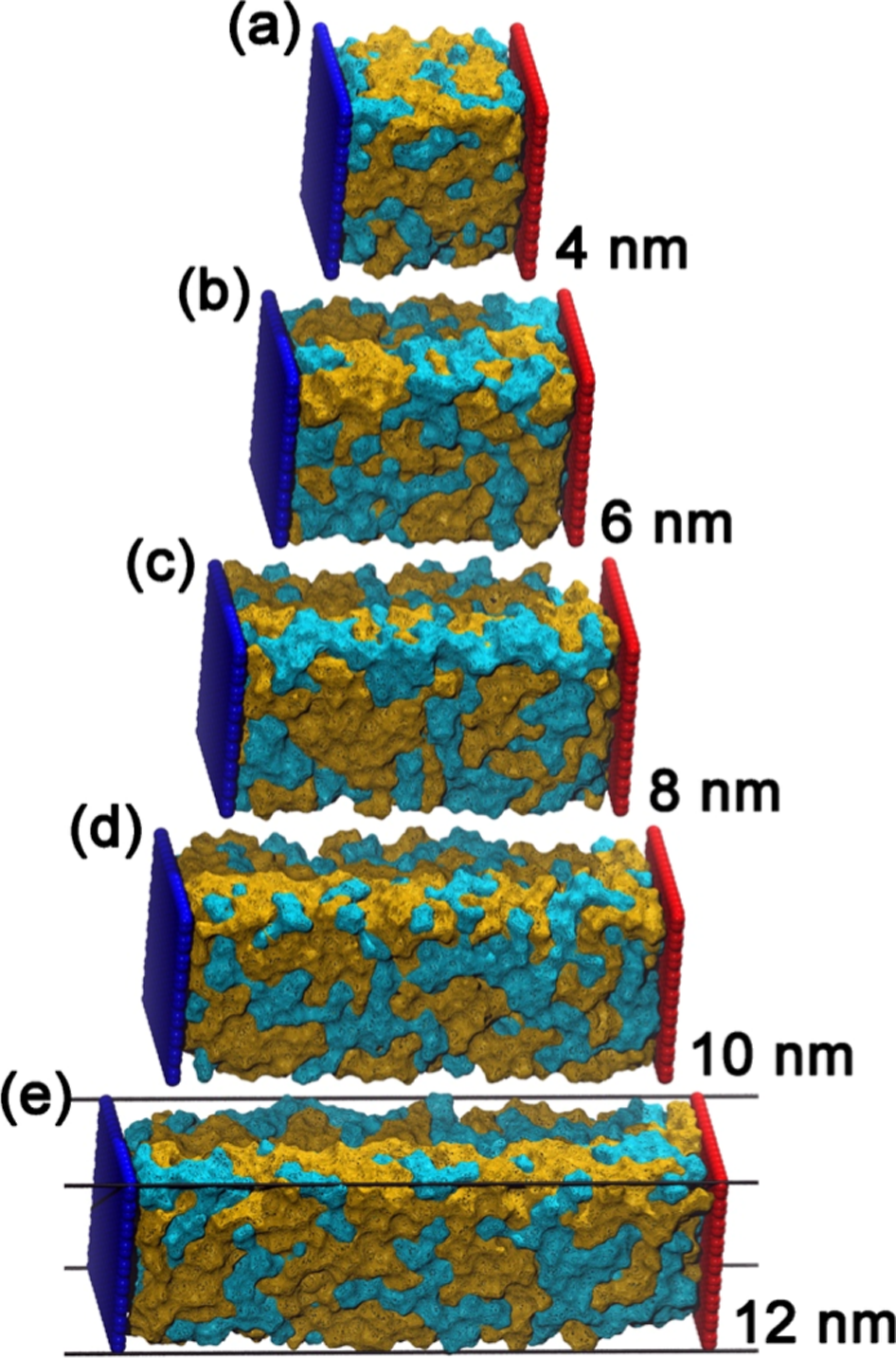
Schematic representation of the five modeled supercapacitor systems with different electrode separations, (a) *L* = 4, (b) *L* = 6, (c) *L* = 8, (d) *L* = 10, and (e) *L* = 12 nm with simulated box limits. The positively and negatively charged graphene electrodes are shown in blue and red, respectively. The electrolyte consists of the ionic liquid [emim]­[ala], with [emim] cations represented in cyan and [ala] anions in orange. These initial configurations were used as the starting point for the molecular dynamics simulations aimed at investigating the effect of electrode spacing on electric double layer (EDL) formation, capacitance, and energy storage.

**Table 1:** Structural and Mass Properties of the Five Simulated Supercapacitor Systems Composed of Graphene Electrodes and the Ionic Liquid [emim]­[ala], with Electrode Separations (*L*) of 4, 6, 8, 10, and 12 nm^a^

*L* (nm)	# IL	volume (×10^–19^ cm^3^)	# atoms electrodes	*m*_IL_ (×10^–19^ g)	*m*_Elect_ (×10^–19^ g)	*m*_Tot_ (×10^–19^ g)	# atoms
12	568	1.7144	1080	1.8800	0.2155	2.0954	18,688
10	471	1.4287	1080	1.5589	0.2155	1.7744	15,681
8	373	1.1429	1080	1.2346	0.2155	1.4501	12,643
6	276	0.8572	1080	0.9135	0.2155	1.1290	9636
4	178	0.5715	1080	0.5891	0.2155	0.8046	6598

a The table lists, for each system, the number of ionic pairs, simulation cell volume (cm3), number of atoms in the electrodes, ionic liquid mass (mIL), electrode mass (mElect), total system mass (mTot), and the total number of atoms. These data establish the baseline configurations employed in the molecular dynamics simulations to investigate the effects of electrode spacing on EDL structure and capacitive behavior.

### 2.2 Simulation Protocol

All molecular dynamics simulations were carried out in the canonical (*NVT*) ensemble at a constant temperature of 500 K, regulated by the v-rescale thermostat^37^ with a coupling constant of 0.1 ps. The choice of 500 K in the present NVT simulations was not intended to reproduce the typical operating temperature of practical supercapacitors. Instead, it was adopted to improve configurational decorrelation and statistical sampling of the highly confined ionic liquid while maintaining a fixed-volume simulation protocol for all electrode separations. In this framework, the average density of each simulated device is fixed by construction, so the role of temperature is mainly to accelerate configurational relaxation rather than to define a complete temperature-dependent thermodynamic state. A rigorous analysis of temperature effects would require a different strategy, in which the electrolyte density is first determined at the target temperature from bulk NPT simulations and then transferred to the confined supercapacitor model. In a recent graphene-based molecular dynamics study^38^ using this protocol, the electric double layer remained largely preserved over the 300–600 K range and the total capacitance varied by less than 13%, indicating that electrostatic interfacial properties are only moderately affected when temperature is treated together with the corresponding density changes. In another recent study,^39^ the effect of temperature on the EmimBF_4_/graphene system was investigated between 300 and 450 K. The authors concluded that despite small changes in the electrolyte structure, the total capacitance is only slightly affected by heating the system. Therefore, the present work should be interpreted as a spacing-dependent study performed at a single, internally consistent simulated state, rather than as a direct prediction of room-temperature device performance. Periodic boundary conditions were applied along the *x*, *y* and *z* directions to mimic an extended planar electrode geometry, while the system was treated using a three-dimensional periodic (3dc) slab geometry to properly account for electrostatic interactions under confinement. Electrostatic interactions were computed using the particle mesh ewald (PME) method,^40^ while van der Waals interactions were modeled through a Lennard-Jones potential with a cutoff radius of 1.2 nm. Bond constraints involving hydrogen atoms were treated with the LINCS algorithm,^41^ which allowed the use of a 1 fs (0.001 ps) integration time step. Each supercapacitor system was first equilibrated for approximately 15 ns, ensuring stability of the ionic liquid and electrode structures, followed by a 50 ns production stage, from which 25,000 configurations were stored for subsequent statistical analyses of both structural and electrochemical properties. This setup guaranteed robust sampling of ion distributions and electrode–electrolyte interactions across all five electrode separations (*L* = 4, 6, 8, 10, and 12 nm).^11,14,26,42,43^

### 2.3 Charge Densities and Electrochemical Properties

The electrode charging process was simulated using the constant charge model,^11,14,21,23,24,38,42^ in which the total surface charge density on each electrode is fixed during the MD trajectory. In this framework, the charges assigned to the individual electrode atoms remain constant throughout the simulation, ensuring that the electrode maintains a prescribed uniform surface charge density. This approach is computationally efficient and allows systematic evaluation of the influence of electrode polarization on ion distribution, potential profiles, and capacitance.^11,14,38,42^ Although it does not account for electronic polarization or charge redistribution at the atomic scale, the constant charge model has been widely adopted in supercapacitor simulations as a robust first-order approximation to capture the main features of EDL formation under confinement. In the present work, four surface charge densities were applied to the graphene electrodes: 0.00, 0.10, 0.20, and 0.30 e/nm^2^. These were implemented by uniformly distributing the desired total charge over the 540 carbon atoms of each electrode. This ensures a homogeneous charge distribution across the electrode surface and symmetric polarization between the positive and negative electrodes. From the 25,000 configurations stored during the production stage, the electrostatic potential profiles were computed by averaging the charge distributions of ions along the *z*-direction.

### 2.4 Electrochemical Properties

The electrostatic potential profile Φ­(*z*) was obtained along the *z*-axis by integrating the local charge density ρ­(*z*) using the one-dimensional Poisson equation.^44^ To ensure a consistent reference point, the raw potential profile was corrected by applying a linear regression to the central (bulk-like) region of the cell and subtracting this fitted line across the full profile. This adjustment guarantees that the potential in the middle of the supercapacitor is set to zero, eliminating artifacts introduced by periodic boundary conditions or dipole asymmetries. The potential drop (ΔΦ) between positively and negatively charged electrodes was then determined by comparing the corrected potentials in the discharged and charged states (Φ_D_ and Φ_C_, respectively). The differential capacitance (*C*_diff_) was evaluated by fitting the variation of potential with respect to surface charge density, i.e., from the slope of the linear regression of Φ_±_ ×σ_±_ , yielding the capacitances of the positive and negative electrodes, *C*_+_ and *C*_–_, respectively. Considering the series configuration of the two electrodes, the total device capacitance was calculated as $C_{Tot}=C_{+}+\frac{C_{-}}{C_{+}+C_{-}}$ . Finally, the energy analysis was carried out using three distinct normalization bases. Because the total capacitance reported here, *C*_Tot_, is expressed per unit electrode area (μF/cm^2^), the projected areal energy density was calculated as $U_{proj}\left(\Delta \Phi \right)=\frac{1}{2}C_{Tot}{\left(\Delta \Phi \right)}^{2}$ . The gravimetric and volumetric energy densities were then computed as $u_{g}=\frac{U}{m_{Tot}}$ and $u_{v}=\frac{U}{V_{device}}$ , respectively, with *U* = *U*_proj_ *A*_device_. Here, *m*_Tot_ = *m*_IL_ + *m*_elect_ corresponds to the total mass of the simulated supercapacitor, including the confined ionic liquid and the two graphene electrodes, and *V*_device_ = *A*_device_ *L* corresponds to the interelectrode device volume listed in Table 1; the external vacuum slab used in the simulation cell was not included in this calculation. Since the present model is planar rather than porous, no pore-volume correction is required. In addition, the areal energy associated with the voltage-dependent capacitance curves shown in Figure 6 was obtained from the thermodynamic relation $U_{\mathrm{int}}=\int_{0}^{\Delta \Phi_{\max}}C_{Tot}\left(\Delta \Phi \right)\Delta \Phi d\left(\Delta \Phi \right)$ . For comparative purposes, ΔΦ_max_ = 2.5 V was adopted as a common upper integration limit for all *L* values. Therefore, areal energy reflects the intrinsic interfacial storage capacity of the graphene/electrolyte interface, whereas gravimetric and volumetric energy densities additionally incorporate architectural contributions through total mass and confined device volume.

## 3 – Results and Discussion

### 3.1 Electric Potential

In the discharged supercapacitors (σ = 0.00 e/nm^2^), the potential values at the electrodes remain practically symmetric, resulting in potential differences (ΔΦ) close to zero (see Table 2). The largest deviation appears at *L* = 8 nm, with ΔΦ = 0.10 V, which corresponds to a difference of only ∼14%relative to the average electrode potentials (≈0.7 V). For the other separations, ΔΦ remains below 0.03 V, indicating that under the point of zero charge (PZC) condition the system is effectively neutral and the average electric field between the electrodes can be considered negligible. These results confirm that the correction applied to the potential profiles was effective in canceling artificial displacements in the center of the simulation cell.

**Table 2:** Electrostatic Potential Values Obtained from the Molecular Dynamics Simulations for the [emim]­[ala]-Based Supercapacitors at Different Electrode Separations (L = 4, 6, 8, 10, and 12 nm) and Surface Charge Densities (σ = 0.00, 0.10, 0.20, and 0.30 e/nm2)^a^

	σ = 0.00 e/n^m2^			σ = 0.10 e/nm^2^			σ = 0.20 e/nm^2^			σ = 0.30 e/nm^2^		
*L*(nm)	Φ_+_	Φ_–_	ΔΦ	Φ_+_	Φ_–_	ΔΦ	Φ_+_	Φ_–_	ΔΦ	Φ_+_	Φ_–_	ΔΦ
4	0.70	0.70	0.00	0.99	0.38	0.61	1.24	–0.04	1.28	1.48	–0.49	1.97
6	0.73	0.70	0.03	1.01	0.37	0.61	1.30	–0.08	1.35	1.52	–0.49	1.98
8	0.77	0.67	0.10	1.04	0.33	0.61	1.21	0.01	1.10	1.51	–0.49	1.91
10	0.71	0.70	0.01	1.04	0.30	0.73	1.25	–0.03	1.27	1.49	–0.55	2.03
12	0.72	0.67	0.05	1.00	0.35	0.60	1.28	–0.06	1.29	1.51	–0.48	1.94

a For each system, the corrected potentials at the positive electrode (Φ+) and negative electrode (Φ−) are reported, together with the resulting potential drop (ΔΦ) across the cell after applying the linear regression adjustment to enforce Φ = 0 at the midpoint of the simulated supercapacitor (in *V*).

When the electrodes are charged up to σ = 0.30 e/nm^2^, a significant increase in the resulting device potential is observed. The value of ΔΦ rises to the range of ∼1.91 to 2.03 V, representing an average increase of approximately 20–40 times compared to the discharged state (σ = 0.00 e/nm^2^). For instance, at *L* = 10 nm, ΔΦ increases from only 0.01 V in the discharged system to 2.03 V in the charged system, a jump of more than 3 orders of magnitude. This behavior directly reflects the strong polarization of the electrodes and the consequent reorganization of the ionic liquid, responsible for the efficient formation of the electric double layer (EDL) under the applied model. It is also noteworthy that the positive electrode exhibits a larger absolute potential compared to the negative electrode (about three times greater), a trend observed for all values of *L*, highlighting the independence of this property from the device length. The analysis of the intermediate charge states, at σ = 0.10 e/nm^2^ and σ = 0.20 e/nm^2^, shows that the growth of ΔΦ is not linear with respect to the surface charge density. From σ = 0.00 to σ = 0.10 e/nm^2^, ΔΦ increases by an average of ∼ 0.6 V, and from σ = 0.10 to σ = 0.20 e/nm^2^ the additional gain is ∼0.6 V. The step from σ = 0.20 to σ = 0.30 e/nm^2^ corresponds to an increase of approximately ∼0.8 V. This trend suggests that the capacitive response of the system depends not only on the amount of imposed charge but also on ionic reorganization and the geometric packing limitations of the confined ionic liquid, which vary with the intensity of the resulting local electric field.

Finally, the dependence of the potential difference (ΔΦ) on the electrode separation (*L*) reveals that, for both discharged and charged states, the values remain relatively stable without a clear monotonic trend. For σ = 0.30 e/nm^2^, ΔΦ varies from 1.94 V (*L* = 12 nm) to 2.03 V (*L* = 10 nm), a difference of ∼5% between the lowest and highest values. In the discharged case (σ = 0.00 e/nm^2^), ΔΦ fluctuations remain below 0.10 V across the entire *L* range. These results indicate that, within the studied regime, the capacitance will be predominantly governed by the imposed surface charge density and the microstructure of the EDL, while the geometric separation between electrodes exerts only a secondary influence on the global potential difference. Despite the close values obtained in this analysis, it can be observed that ΔΦ decreases as *L* increases, except at *L* = 10 and 12 nm, where a persistent outlier value disrupts the ΔΦ *vs*. *L* decay pattern for *L* < 10 nm. These results for *L* = 10 and 12 nm were checked and confirmed in additional simulations. Figure 2 shows the electric potential profile inside the supercapacitor for the discharged (charged) state and highlights, for each case, the raw ΔΦ obtained between the electrodes.

**Figure 2:**
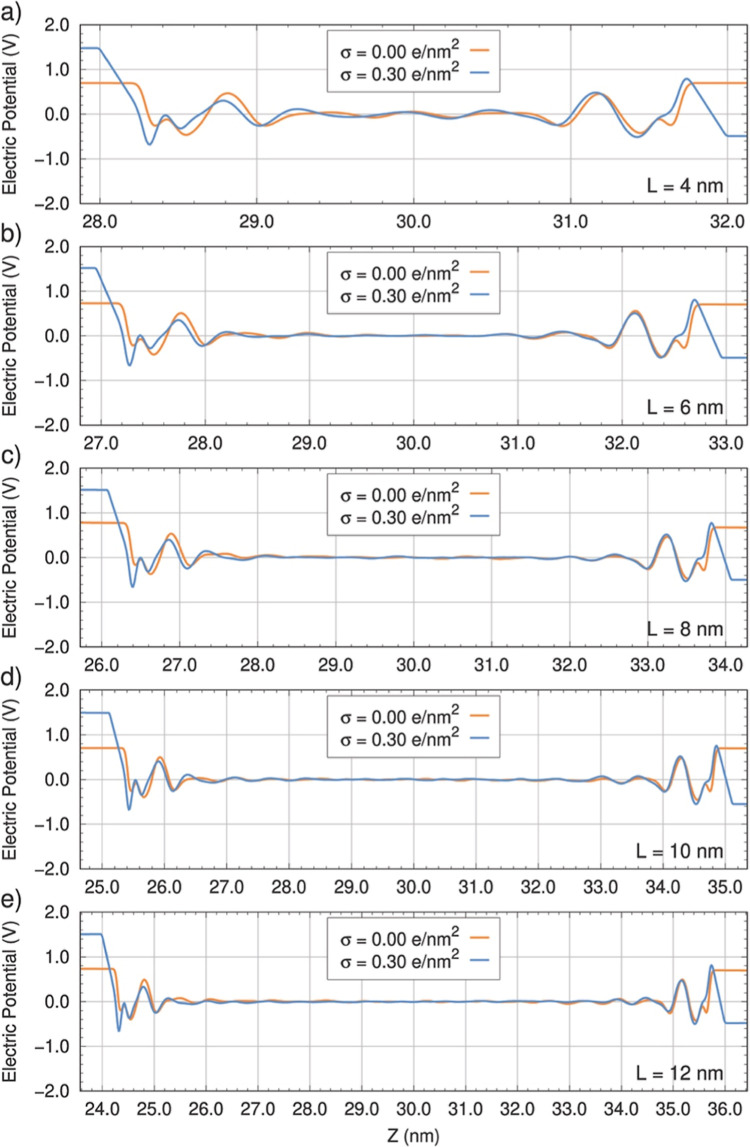
Electric potential profiles for the charged and discharged systems of all simulated supercapacitor models. In each panel, the charged state (σ = 0.30e/nm^2^) is represented by the cyan curve, while the discharged state (σ = 0.00e/nm^2^) is represented by the orange curve. (a) *L* = 4, (b) *L* = 6, (c) *L* = 8, (d) *L* = 10, and (e) *L* = 12 nm.

### 3.2 Mass Density Profile and Electric Double Layer

The mass density profiles (see Figure 3) obtained for the ionic liquid [emim]­[ala] at different electrode separations (*L* = 12, 10, 8, 6, and 4 nm) clearly show the systematic formation of structured ionic layers adjacent to the charged surfaces. Regardless of the value of L, well-defined adsorption peaks are observed, characterizing the formation of the electric double layer (EDL), a phenomenon expected for systems under high polarization (σ = 0.30 e/nm^2^). The graphene electrodes (represented by the blue and red regions) delimit the confinement, and the behavior of the curves shows that the intensity of these peaks varies with the electrode separation.

**Figure 3:**
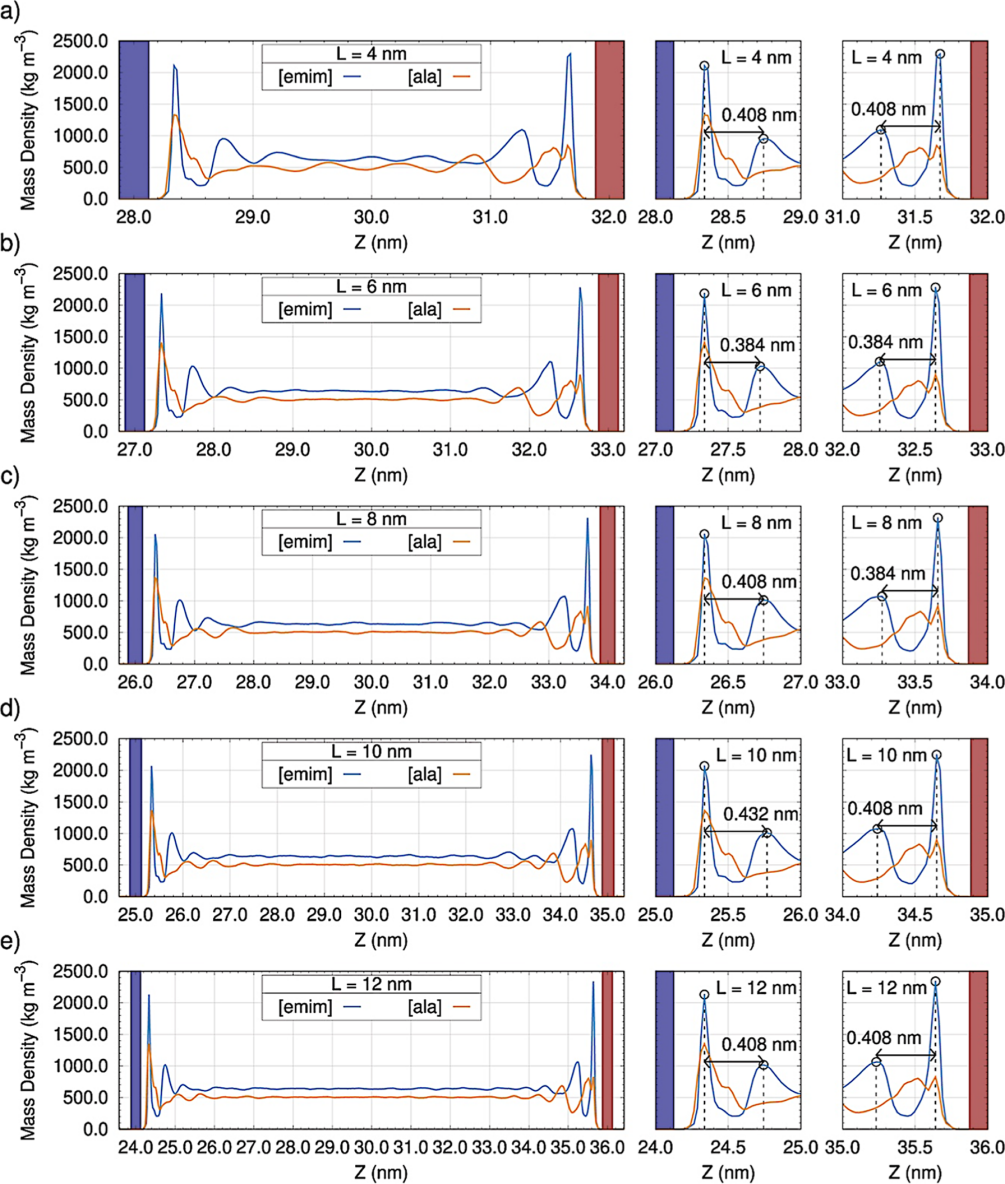
Mass density profiles (ρ, in kg/m^3^) of the ionic liquid [emim]­[ala] confined between graphene electrodes at different electrode separations, (a) *L* = 4, (b) *L* = 6, (c) *L* = 8, (d) *L* = 10, and (e) *L* = 12 nm, under high polarization (σ = 0.30 e/nm^2^). The blue and red regions correspond to the positively and negatively charged electrodes, respectively. The density profiles of [emim] cations and [ala] anions are shown in cyan and orange, respectively, across the *z* direction of the simulation box. For large separations (*L* ≥ 10 nm), a bulk-like central region is preserved, whereas for shorter distances (*L* ≤ 6 nm) strong overlap of the electric double layers (EDLs) occurs, eliminating the homogeneous zone and leading to pronounced oscillations throughout the confined channel. The right-hand panels show zoomed-in EDL profiles used to estimate the effective EDL thickness.

The analysis of the dependence on electrode spacing L reveals significant differences between wide and confined systems. For *L* = 12 and 10 nm, a homogeneous central region is present, where the total density remains stable around 1.0–1.2 g/cm^3^, indicating the preservation of bulk-like behavior. In contrast, for *L* = 8, 6, and 4 nm, density oscillations originating from each electrode strongly overlap, eliminating the flat central region. This behavior is particularly evident at *L* = 4 nm, where no free “bulk-like” volume is observed; instead, a continuous modulation of density is present throughout the confined space. At the ionic level, the distribution of the ionic liquid components is selective and consistent with the applied polarization. The [emim] cations accumulate preferentially near the negative electrode, while the [ala] anions concentrate near the positive electrode. As confinement increases, the adsorption peaks become more intense and narrower, reflecting stronger ionic correlation and packing restrictions under intense electric fields. This phenomenon contributes to the easy enhancement of capacitance at short interelectrode distances, but it can also lead to limitations in ionic mobility, thereby affecting the dynamic response of the device.

The right side of Figure 3 presents a zoomed-in EDL mass–density profile, where the effective EDL thickness was estimated by measuring the distance between the first two successive peaks of the same electrolyte component. The [emim] cation was used for this purpose because its peaks are more pronounced due to its larger molar mass compared to the anion [ala]. Near the positively charged electrode, a higher concentration of [ala] anions is observed, accompanied by [emim] cations, forming an EDL with a effective thickness ranging from 0.384 to 0.432 nm. In contrast, at the negatively charged electrode, a larger accumulation of [emim] molecules occurs directly at the surface, pushing the [ala] anions toward the interior of the device and producing a clearer alternation of electrolyte components, resulting in a slightly more well-defined EDL with effective thickness between 0.384 and 0.408 nm. This asymmetry indicates that the EDL is somewhat more extended at the positive electrode, reflecting both the intrinsic differences between cations and anions and the geometry and charge distribution of the graphene atoms. The influence of electrode spacing (*L*) is also evident: for systems with *L* ≥ 10 nm, the EDLs remain well-defined and independent, separated by a central region with bulk-like behavior, whereas for *L* ≤ 4 nm a strong overlap between the layers occurs, eliminating the homogeneous central region and intensifying density oscillations throughout the channel. Based on these estimates, the EDL occupies a significant fraction of the simulated device, corresponding to approximately 20% of the channel for *L* = 4 nm, 13% for *L* = 6 nm, 10% for *L* = 8 nm, 8% for *L* = 10 nm, and 7% for *L* = 12 nm. These results highlight that the EDL microstructure and its effective thickness depend not only on polarization but also on the degree of confinement imposed by the system. It should also be noted that, in the preparation of the initial systems, the number of ionic pairs in each confinement volume was calculated to maintain the same overall density.

The counting of particles adsorbed in the regions adjacent to the electrodes (see Table 3) and in the interior of the supercapacitor was performed considering 5000 configurations extracted from the MD trajectory. This statistical sampling strategy ensures that the reported values correspond to representative averages of the system’s behavior over the simulation time, enabling consistent analyses of ionic structuring under different confinement conditions. The analysis of the average ionic population in the layers near the electrodes (up to 1 nm from the surface) highlights the typical selectivity of electric double layer (EDL) formation in supercapacitors. For all average values obtained across the different *L*, it is observed that [ala] anions predominate near the cathode, while [emim] cations are more concentrated at the anode, in accordance with the applied polarization. The average number of particles in the EDL is significant, ranging from ∼32 to ∼45 ions per species at each electrode, with a trend of intensified adsorption as confinement increases. This behavior confirms that ionic structuring is strongly driven by the local electric field, promoting the selective accumulation of oppositely charged species at the graphene surface.

**Table 3:** Average Number of [Emim] Cations and [ala] Anions Adsorbed in the Electric Double Layers (EDLs, up to 1 nm from the Electrode Surfaces) and in the Central Bulk Region (Particles/nm) of the Simulated Supercapacitors, for Different Electrode Separations (*L* = 12, 10, 8, 6, and 4 nm)^a^

*L* (nm)	EDL positive		EDL negative		bulk region	
	# [emim]	# [ala]	# [emim]	# [ala]	# [emim]	# [ala]
4	39	43	45	34	47	50
6	41	44	42	33	48	50
8	35	39	49	37	48	50
10	32	37	50	39	49	49
12	39	43	45	35	48	49

a Values represent averages obtained from 5000 molecular dynamics configurations. The results highlight the selective accumulation of anions near the cathode and cations near the anode, while the bulk ionic density remains stable at ∼(48–50) ions per nm, independent of *L*.

On the other hand, the average number of ions in the central region of the supercapacitor (bulk) shows remarkable stability, with values close to 48–50 ions of each type per nm across all investigated electrode separations *L*. This result indicates that system preparation ensured a globally homogeneous ionic density, regardless of the degree of confinement. Maintaining this constant density is essential to ensure that the observed differences in the EDLs effectively arise from the variation in electrode spacing and not from fluctuations in the total ion concentration. Thus, the bulk serves as a reference, allowing a clear distinction between purely structural effects induced by confinement and density variations. Finally, the influence of *L* on the average composition of the EDLs is evident. For *L* = 12 and 10 nm, the average number of adsorbed ions remains moderate, consistent with the presence of a well-defined bulk-like central region. However, as *L* decreases to 8, 6, and 4 nm, the overlap of the EDLs intensifies local adsorption: at *L* = 6 nm, for example, averages of 41 [emim] cations and 44 [ala] anions are counted near the positive electrode, values significantly higher than those observed at larger separations. In the extreme case of *L* = 4 nm, practically the entire confined region becomes dominated by the electrostatic influence of the EDLs, so that the concept of an independent bulk no longer applies. In this regime, density oscillations span the entire channel, and the average number of adsorbed particles becomes comparable to that observed in the bulk of wider systems. This trend indicates that, under intense confinement, the balance between ionic selectivity, layer-to-layer correlation, and volume restriction results in a highly organized microstructure, in which the EDL ceases to be a localized entity and instead becomes a phenomenon permeating the entire device volume. This result is in full agreement with the mass density profiles (Figure 3), reinforcing that the average electrolyte microstructure is governed not only by surface polarization but also by the degree of geometric confinement.

### 3.3 Capacitance

The determination of capacitance in the modeled supercapacitors was performed using the linear relationship between surface charge density (σ) and the electric potential difference (ΔΦ) obtained during MD simulations. For each system, the set of Φ values as a function of σ was fitted by a linear regression of the form *y* = α*x* + β. When this expression is compared with the fundamental relationship between charge and potential, it becomes evident that the slope coefficient α of the linear regression corresponds to the inverse of the differential capacitance associated with each electrode. Thus, the individual capacitances were determined according to *C* = 1/α, distinguishing the values relative to the positive electrode (*C*_+_) and the negative electrode (*C*_–_). From the values of *C*_+_ and *C*_–_, it was then possible to calculate the equivalent capacitance (*C*_Tot_) of the device, considering the series connection of the two electrodes, according to the relation *C*_Tot_ = (*C*_+_ × *C*_–_)/(*C*_+_ + *C*_–_). This procedure ensures that the results obtained are consistent with the physical model of a supercapacitor, in which the electrodes act as plates of an equivalent capacitor association. Furthermore, to validate the robustness of the method, a third-degree polynomial equation describing the overall behavior of the capacitance as a function of charge density was also fitted, with coefficients adjusted from the simulated values. All fits showed a coefficient of determination *R* ^2^ > 0.99, ensuring excellent statistical quality. It should be emphasized that the capacitance values reported in Table 4 are regression-derived quantities rather than single-point estimates. The values of *C*_+_ and *C*_–_ were obtained from linear fits of electrode potential as a function of surface charge density, and the total capacitance *C*_Tot_ was then calculated from the corresponding series relation. In all cases, the linear regressions yielded coefficients of determination *R* ^2^ > 0.99, indicating very low dispersion of the simulated data around the fitted trends. Therefore, the small variation of *C*_Tot_ across the investigated *L* range should be interpreted in the context of highly consistent regression behavior, as also evidenced by the limited scatter of the data shown in Figure 4.

**Table 4:** Linear Regression Values (Φ­(σ) = *A*σ + *B*) for Positive Electrode Capacitance (*C*_+_), Negative Electrode Capacitance (*C*_–_), and Total Capacitance *C*_Tot_ (μF/cm^2^) for Supercapacitors with *L* Values Equal 4–12 nm^a^

*L* [nm]	*C*_+_	*C*_–_	*C*_Tot_	σ(Φ) and $C_{diff}=\frac{\partial \sigma }{\partial \Phi }$
4	6.17	4.02	2.43	σ(Φ) ≅ 0.177Φ^3^ + 1.168Φ^2^ + 5.169Φ – 0.010
				*C*_diff_(Φ) ≅ 0.531Φ^2^ + 2.337Φ + 5.169
6	6.02	3.98	2.40	σ(Φ) ≅ 0.425Φ^3^ + 1.180Φ^2^ + 4.787Φ + 0.064
				*C*_diff_(Φ) ≅ 1.274Φ^2^ + 2.361Φ + 4.787
8	6.69	4.20	2.58	σ(Φ) ≅ 0.120Φ^3^ + 1.104Φ^2^ + 5.271Φ + 0.319
				*C*_diff_(Φ) ≅ 0.360Φ^2^ + 2.209Φ + 5.271
10	6.22	3.94	2.41	σ(Φ) ≅ 0.388Φ^3^ + 1.280Φ^2^ + 4.860Φ – 0.001
				*C*_diff_(Φ) ≅ 1.165Φ^2^ + 2.560Φ + 4.860
12	6.07	4.14	2.46	σ(Φ) ≅ 0.362Φ^3^ + 1.081Φ^2^ + 4.873Φ + 0.122
				*C*_diff_(Φ) ≅ 1.087Φ^2^ + 2.161Φ + 4.873

a The polynomial representation of σ­(Φ) and its derivative *C*_diff_(Φ) = ∂σ/∂Φ for differential capacitance are shown.

**Figure 4:**
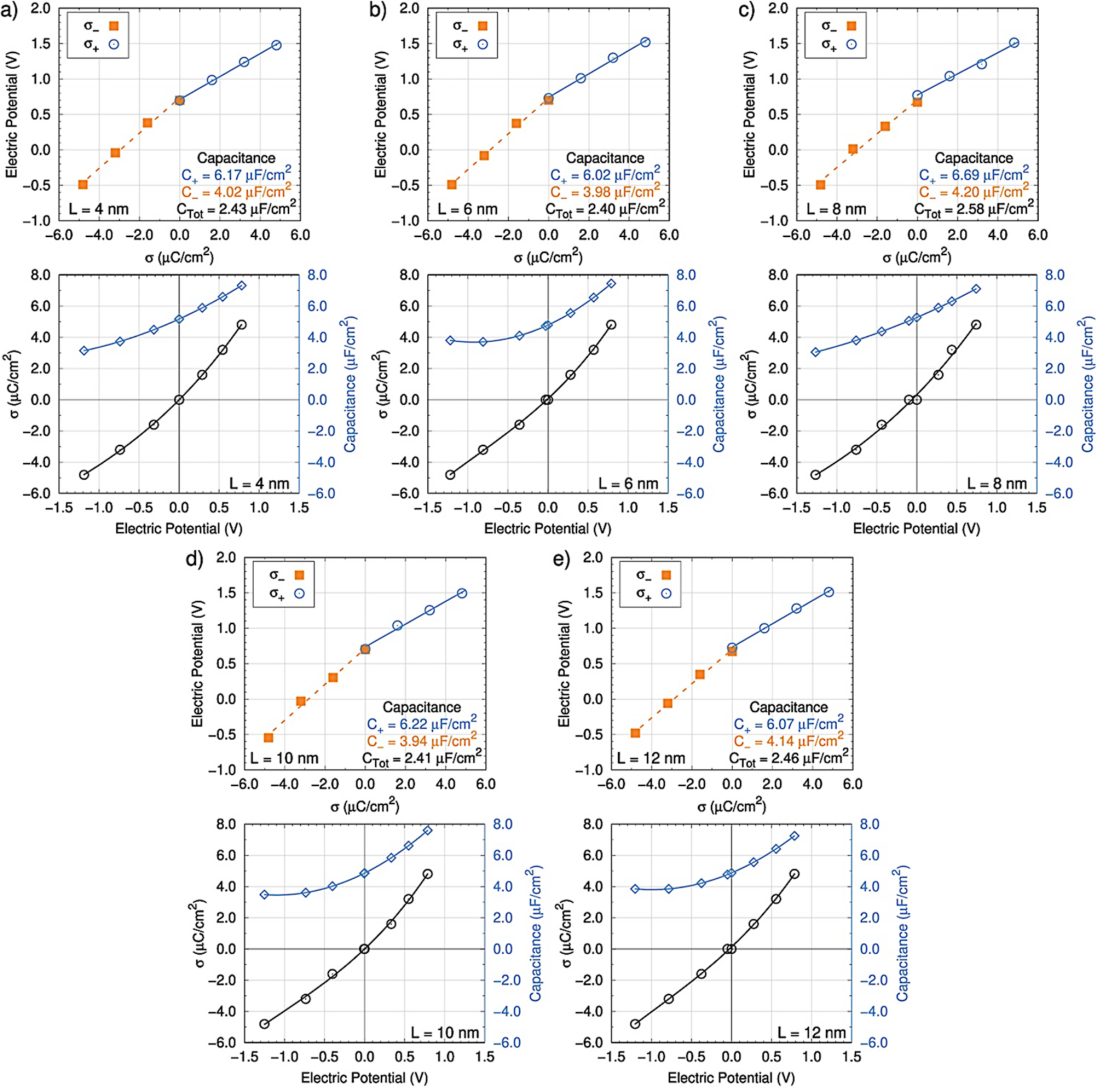
Electric potential (Φ) as a function of surface charge density (σ) and corresponding differential capacitance behavior for different electrode separations: (a) *L* = 4 nm, (b) *L* = 6 nm, (c) *L* = 8 nm, (d) *L* = 10 nm, and (e) *L* = 12 nm. In the upper panels, data for the positive and negative electrodes are shown as orange squares (σ_+_) and blue circles (σ_–_), respectively; the corresponding linear regressions are used to obtain the projected capacitances *C*_+_, *C*_–_, and the equivalent device capacitance *C*_Tot_. In the lower panels, σ­(Φ) is shown together with the polynomial fits used to determine the differential capacitance (*C*_diff_) as a function of potential (Φ). The comparison highlights the asymmetry between positive and negative electrodes and the influence of electrode separation on the overall capacitive response. The capacitance values reported here are regression-derived quantities obtained from linear fits of Φ­(σ), all with *R* ^2^ > 0.99, while the polynomial representation of σ­(Φ) and its derivative *C*_diff_(Φ) = ∂σ/∂Φ is used to describe the differential capacitance behavior.

The obtained values show that the individual electrode capacitances range from approximately 6.02–6.69 μF/cm^2^ for *C*_+_ and 3.94–4.20 μF/cm^2^ for *C*_–_, with the negative electrode systematically exhibiting a lower charge storage capacity. This asymmetry reflects differences in the structural organization of the electrolyte near the positive and negative surfaces, as previously evidenced by the mass density and ionic distribution analyses. As a consequence, the equivalent capacitance *C*_Tot_ of the device presents slightly different values, varying between 2.40 μF/cm^2^ (*L* = 6 nm) and 2.58 μF/cm^2^ (*L* = 8 nm), reflecting the limitation imposed by the structure. The comparison between different electrode separations (*L*) reveals that *C*_Tot_ does not exhibit large variations, but rather fluctuates within a relatively narrow range. This oscillatory behavior suggests that the electrolyte microstructure, modulated by the partial or total overlap of the electric double layers (EDLs), exerts a modest influence on the capacitive response of the device. Finally, the third-degree polynomial fits presented in Table 4 provide a more comprehensive description of the capacitance behavior as a function of the applied charge. These polynomials reproduce the observed nonlinear trends, capturing subtle variations that are not described by simple linear regression. Although linear regression is sufficient to obtain the average differential capacitance values (*C*_+_, *C*_–_, and *C*_Tot_), the polynomial approach allows the analysis to be extended to broader charge conditions, in which the system response may deviate from a strictly linear regime. Thus, the results confirm that capacitance in confined ionic supercapacitors is a phenomenon sensitive to both surface polarization and the degree of structural confinement of the device, requiring multiscale analyses for a complete description. Based on the obtained results, Figure 4 illustrates the dependence on *L* of the projected capacitance (obtained from the linear regression of electric potential as a function of charge density in the device) as well as the differential capacitance derived from the polynomial fits presented in Table 4. Additionally, when the exact zero-charge condition σ = 0.00 e/nm^2^ is observed, the differential capacitance at the corresponding potential Φ_PZC_ can be calculated by $C_{diff}=\frac{\partial \sigma }{\partial \Phi }$ . The resulting values remain confined to a relatively narrow range, approximately 2.38–2.59 μF/cm^2^ for *L* between 4 and 12 nm. This limited variation indicates that the differential capacitance at the uncharged state is weakly dependent on electrode separation within the investigated confinement regime. Therefore, the zero-charge capacitance appears to be primarily controlled by the intrinsic interfacial properties of the graphene electrodes rather than by geometric effects associated with device length.

### 3.4 Energy

The projected energy density results (Figure 5), obtained from $U=\frac{1}{2}C_{Tot}{\left(\Delta \Phi \right)}^{2}$ and fitted with a quadratic model of the form *y* = α*x* ^2^, confirm that the device operates in a predominantly electrostatic regime over the investigated potential window. Both gravimetric (*U*/*m*) and volumetric (*U*/*V*) energy densities exhibit a strictly quadratic dependence on the applied potential difference, with no detectable linear contribution, which is consistent with ideal EDL supercapacitor behavior. This reinforces the conclusion drawn from the capacitance analysis that the charge storage mechanism is dominated by interfacial polarization at the graphene-electrolyte interface rather than by Faradaic processes. Quantitatively, the gravimetric energy density increases significantly with (ΔΦ), as expected from the quadratic scaling. At ΔΦ = 2.0 V, the estimated *U*/*m* values are approximately 8.7 J/g (4 nm), 6.0 J/g (6 nm), 4.8 J/g (8 nm), 3.8 J/g (10 nm), and 3.3 J/g (12 nm). At 1.5 V, the corresponding values are approximately 4.9, 3.4, 2.7, 2.2, and 1.9 J/g, respectively. Similarly, the volumetric energy density at 2.0 V reaches approximately 12.3 J/cm^3^ (4 nm), 7.9 J/cm^3^ (6 nm), 6.1 J/cm^3^ (8 nm), 4.8 J/cm^3^ (10 nm), and 4.0 J/cm^3^ (12 nm). At 1.5 V, these values are approximately 6.9, 4.5, 3.4, 2.7, and 2.3 J/cm^3^, respectively. These magnitudes are consistent with high-performance graphene-based supercapacitor architectures operating within a 2 V window.

**Figure 5:**
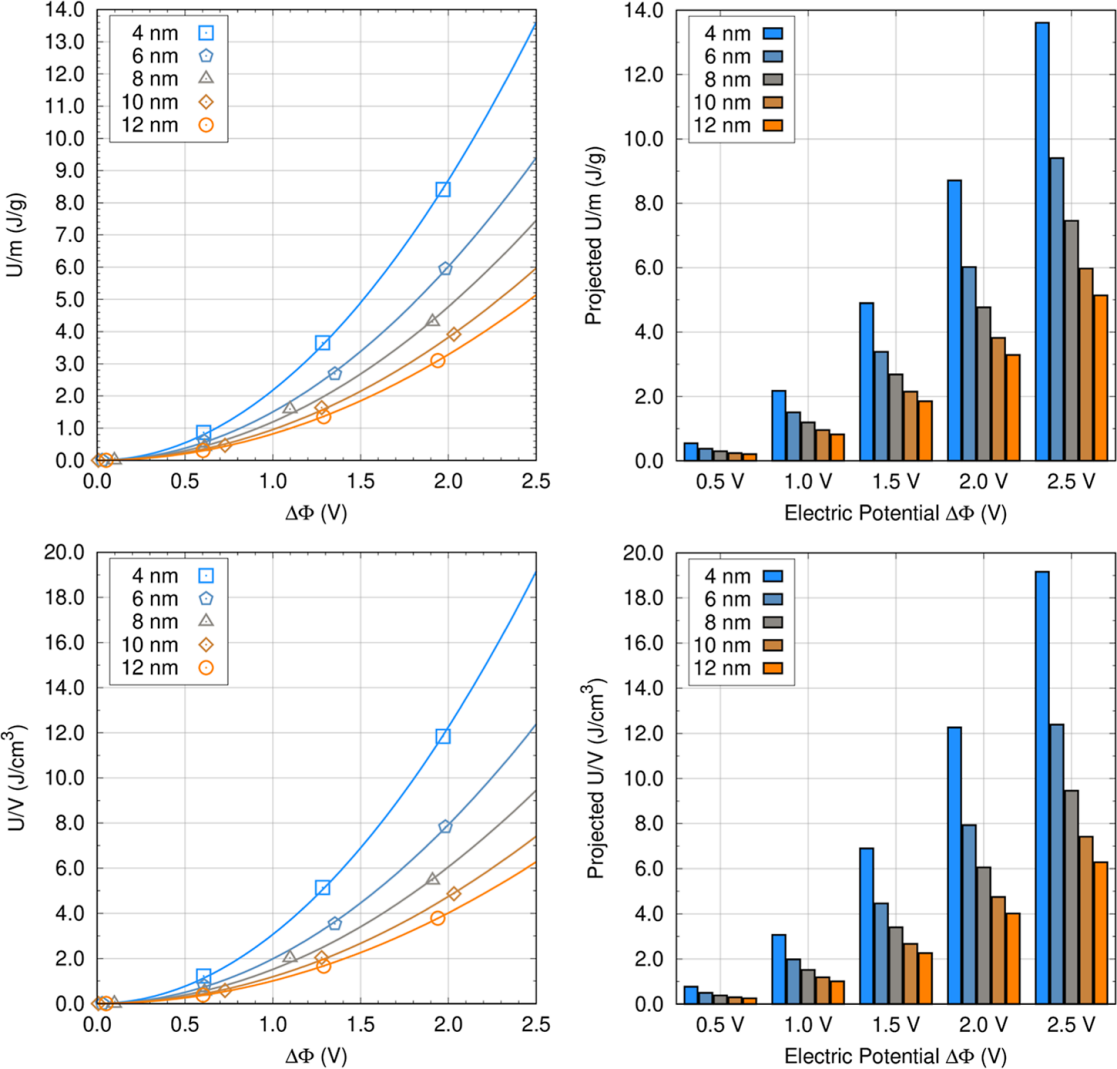
Gravimetric (*U*/*m*) and volumetric (*U*/*V*) energy densities of the graphene-based supercapacitor as a function of the applied potential difference ΔΦ for electrode separations *L* = 4, 6, 8, 10, and 12 nm. Left panels: continuous curves represent quadratic fits of the form $U=\frac{1}{2}C_{Tot}{\left(\Delta \Phi \right)}^{2}$ , projected from the total capacitance values, with symbols indicating the calculated data points. Right panels: bar charts showing the projected energy densities at selected operating voltages (0.5, 1.0, 1.5, 2.0, and 2.5 V). The strictly quadratic dependence confirms the electrostatic double-layer storage mechanism, while the systematic decrease in energy density with increasing *L* reflects architectural effects on mass and volume normalization rather than substantial changes in total capacitance.

A clear dependence on the electrode separation *L* emerges when energy density is analyzed, even though the total capacitance itself varies only weakly with *L*. Devices with smaller separations (e.g., *L* = 4 nm) consistently display the highest gravimetric and volumetric energy densities, while larger separations (*L* = 12 nm) yield systematically lower values. Importantly, this trend does not arise from large changes in *C*_Tot_, which remains nearly constant across 4–12 nm. Instead, the reduction in energy density with increasing *L* reflects the normalization by mass and volume. As *L* increases, the amount of electrolyte (and thus mass and volume) grows proportionally, while the effective interfacial capacitance remains approximately constant. Consequently, the stored energy per unit mass or volume decreases. This observation highlights a key design principle for confined graphene-based supercapacitors: within the studied regime, the charge storage process is interfacial and largely independent of geometric spacing, but the energy density performance metrics are strongly influenced by device architecture. Minimizing electrode separation enhances both gravimetric and volumetric energy densities (geometric effect), not because it dramatically increases capacitance, but because it reduces inactive mass and volume contributions. Therefore, optimization strategies aimed at improving energy density should prioritize architectural compactness and maximization of the electrochemically active surface area, while also expanding the electrochemical stability window, since the quadratic dependence on ΔΦ implies that increasing the operational voltage has a significantly larger impact on energy density than modest variations in capacitance.

The five panels (Figure 6) present, for each electrode separation *L*, two independent reconstructions of the total capacitance as a function of the applied potential difference, *C*_Tot_(ΔΦ): (i) values directly obtained from simulation with PZC correction and (ii) capacitance reconstructed from the differential capacitance formalism, followed by a functional fit in ΔΦ. In both approaches, the stored energy was calculated through the thermodynamically consistent expression $U=\int_{0}^{\Delta \Phi_{\max}}C_{Tot}\left(\Delta \Phi \right)\Delta \Phi d\left(\Delta \Phi \right)$ , with ΔΦ_max_ = 2.5 V. The shaded areas in each panel represent the integrated energy density per unit area. For *L* = 4 nm, the integrated energies are 7.71 μJ/cm^2^ (direct simulation) and 7.09 μJ/cm^2^ (reconstructed from *C*_diff_), corresponding to a deviation of approximately 8%. For *L* = 6 nm, the values are 7.42 μJ/cm^2^ and 7.71 μJ/cm^2^, respectively (∼3.9% difference). At *L* = 8 nm, the energies are 7.47 μJ/cm^2^ (direct) and 6.84 μJ/cm^2^ (reconstructed), again differing by about 8%. For *L* = 10 nm, the agreement is nearly exact (7.39 μJ/cm^2^ versus 7.42 μJ/cm^2^, <1% difference), while for *L* = 12 nm the values are 7.53 μJ/cm^2^ and 7.75 μJ/cm^2^ (∼2.9% difference). Overall, discrepancies between the two independent methodologies remain within a few percent and show no systematic trend with increasing *L*.

**Figure 6:**
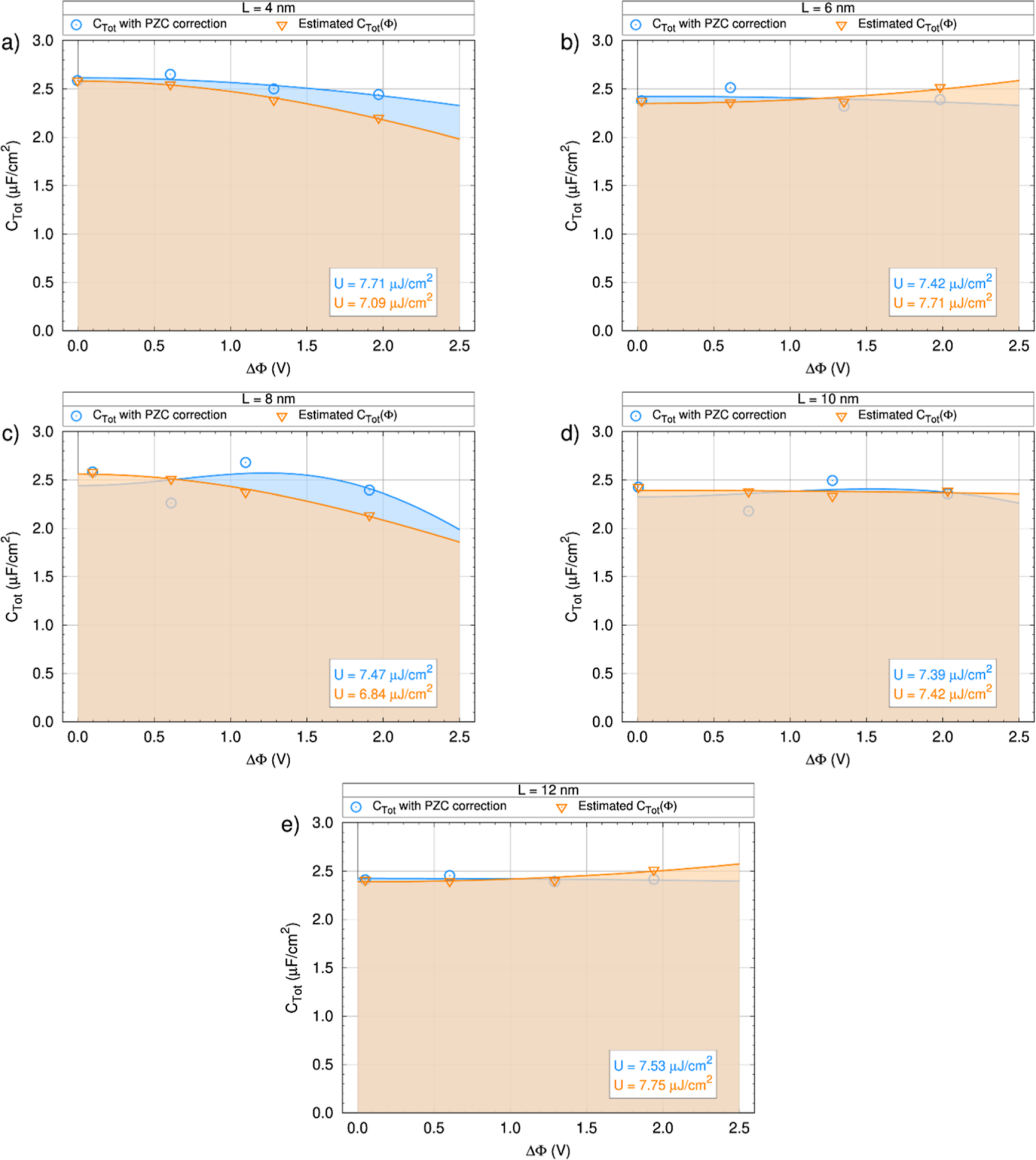
Total capacitance *C*_Tot_(ΔΦ) and corresponding areal energy density for graphene-based supercapacitors with electrode separations (a) 4 nm, (b) 6 nm, (c) 8 nm, (d) 10 nm, and (e) 12 nm. Blue circles represent the simulation results obtained directly from the PZC-corrected data, whereas orange triangles correspond to values reconstructed from the differential-capacitance approach; solid lines are quadratic fits. The shaded regions represent the integrated energy $U=\int_{0}^{\Delta \Phi_{\max}=2.5V}C_{Tot}\left(\Delta \Phi \right)d\left(\Delta \Phi \right)$ .

More importantly, the total stored energy per unit area remains remarkably stable across the entire 4–12 nm range. The directly computed values cluster tightly between 7.39 and 7.71 μJ/cm^2^, while the reconstructed values range between 6.84 and 7.75 μJ/cm^2^. This narrow spread indicates that the integrated energy density is essentially insensitive to electrode separation within the investigated confinement regime. The small variations observed are primarily associated with differences in the curvature of *C*_Tot_(ΔΦ) arising from the fitting procedure rather than with intrinsic geometric effects. This behavior is consistent with a surface-dominated electrochemical double-layer mechanism. Since charge storage occurs at the graphene-electrolyte interface, the effective capacitance (and therefore the integrated areal energy) remains largely independent of the geometric gap between electrodes. The integral formulation further reduces sensitivity to local nonlinearities in *C*_Tot_(ΔΦ), yielding a robust estimate of the total stored energy. While gravimetric (*U*/*m*) and volumetric (*U*/*V*) energy densities exhibit a clear dependence on *L* due to normalization by mass and volume, the total areal energy density does not show a meaningful dependence on electrode separation. This reinforces the interpretation that, within the 4–12 nm regime, the supercapacitor operates in an interfacial regime where the overall energy storage capability per unit area is governed by electric double-layer formation at graphene surfaces rather than by the macroscopic device geometry.

## 4 Conclusions

This study systematically evaluated the influence of electrode separation (*L*) on the structural and electrochemical behavior of graphene-based supercapacitors containing the ionic liquid [emim]­[ala] through classical molecular dynamics simulations. Structural analyses revealed that decreasing *L* intensifies electric double-layer (EDL) overlap, progressively eliminating the bulk-like central region and promoting pronounced ionic layering across the entire confined channel. Particle counting and mass density profiles confirmed enhanced ionic adsorption and correlation under strong confinement, while corrected electric potential profiles demonstrated consistent electrostatic behavior across all systems.

Despite these substantial microstructural differences, the total device capacitance remains nearly constant (∼2.38–2.59 μF/cm^2^), and the differential capacitance at zero charge exhibits only minor variation throughout the 4–12 nm range. The integrated areal energy density up to 2.5 V (∼7.4–7.7 μJ/cm^2^) is likewise essentially independent of *L*. These results demonstrate that charge storage is predominantly governed by interfacial graphene-electrolyte interactions rather than by geometric spacing, confirming a surface-dominated electrostatic storage mechanism within the investigated confinement regime. In contrast, gravimetric and volumetric energy densities decrease systematically with increasing *L* due to mass and volume normalization effects. At 2.0 V, the gravimetric energy density decreases from ∼8.7 J/g (4 nm) to ∼3.3 J/g (12 nm), while volumetric energy density decreases from ∼12.3 to ∼4.0 J/cm^3^. Thus, although intrinsic capacitance and areal energy remain stable, architectural compactness directly enhances practical performance metrics.

These findings have direct implications for microsupercapacitors and on-chip energy storage devices, where maintaining high areal energy while minimizing thickness is critical. Reducing electrode spacing enables lighter and thinner architectures without compromising intrinsic capacitive behavior, which is particularly advantageous for integrated electronics, Internet-of-Things systems, and miniaturized sensors. Similarly, in flexible and wearable technologies, where weight and form factor strongly constrain design, optimizing *L* offers a structural route to improve gravimetric and volumetric energy densities without altering electrolyte chemistry. Beyond portable electronics, the results are also relevant for lightweight energy storage in drones and small electric vehicles, where reductions in mass directly translate into improved operational efficiency. Finally, this work highlights the importance of treating electrode spacing as a rational design variable in computational optimization frameworks. By decoupling interfacial electrochemistry from architectural geometry, molecular simulations can be employed to prescreen nanogap configurations, guiding experimental design toward compact, high-energy-density supercapacitors with predictable performance.
